# Learning ideals: Critical reflections on a near-peer initiative at Leeds

**DOI:** 10.15694/mep.2017.000157

**Published:** 2017-09-13

**Authors:** Alison Ledger, Lydia Edwards, Drew Harding, Ken Hargreaves, Sonal Mistry

**Affiliations:** 1 Leeds Institute of Medical Education; 2 Northwick Park Hospital

**Keywords:** Near-peer learning, Peer-assisted learning, Medical education intercalation

## Abstract

This article was migrated. The article was marked as recommended.

Previous definitions of peer-assisted learning portray the peer-teacher as a non-expert in teaching content and delivery. In this paper, we reflect on a near-peer initiative at our medical school which seems to depart from this definition. This initiative involves intercalating medical education students in the delivery of foundational sessions on professionalism for first year students for a full year, with individual supervision and support from an experienced teacher and extended medical education study. Reflections from a range of people involved are brought together to begin to understand the supportive features and challenges of near-peer teaching in our context and to identify areas for future research. These reflections highlight the potential for differences and contradictions in the ways that teachers and learners are understood within peer-assisted learning initiatives, and emphasize the need to consider the teaching context in peer-assisted learning scholarship.

## Introduction

A number of reviews and guides have reported benefits of peer-assisted learning (PAL) for tutors, tutees, and institutions (for examples, see Irvine et al., 2017; Ross & Cameron, 2007; Tai et al., 2016b). Most previous PAL scholarship has taken the form of standard teaching evaluations, reporting satisfaction ratings or assessing students’ later performance in tests of knowledge or skill ( Irvine et al., 2017; Tai et al., 2016b). This means that gains in clinical knowledge and skills are most frequently reported, whereas outcomes in harder to measure areas, such as professionalism, remain more speculative ( Tai et al., 2016a; 2016b).

PAL scholarship is also criticised for a lack of theoretical frameworks ( Irvine et al., 2017) and previous authors have expressed concern that PAL is often reduced to an “efficient and effective means of knowledge transfer” ( Tai et al., 2016a, p. 674). To date, benefits of PAL are most frequently explained with reference to notions of cognitive and social congruence (for examples see Ross & Cameron, 2007; Stephens et al., 2016), the idea that peers or near-peers’ closeness to learners allows them to better empathise and express themselves in ways that can be readily understood ( Cianciolo et al., 2016). However, a more detailed understanding of PAL is beginning to emerge through studies which have employed observational methods ( Cianciolo et al., 2016; Tai et al., 2016a) or socio-cultural theoretical frameworks ( Bennett et al., 2015). These studies have drawn greater attention to the complexities and tensions which may arise when PAL initiatives are introduced in cultures that traditionally value expertise and learning for the exam. Further reflection on culture and context is necessary to move beyond simple understandings of PAL and maximise peer-to-peer and near-peer learning. This paper aims to contribute such a reflection.

## Background

In the UK, where we are based, medical schools are required to develop curricula which support students’ development as teachers and lifelong learners. The General Medical Council ( GMC, 2015) expects that medical school graduates are able to “reflect, learn, and teach others” (p. 8) and “teaching, training, supporting, and assessing” are among the standards expected of doctors ( GMC, 2014, pp. 14-15). Here at the University of Leeds, undergraduate medical students have frequent opportunities to develop their teaching skills, through required and selected course components as well as voluntary participation in teaching courses and societies. One option available to medical students is our intercalated degree in medical education, the BSc in Applied Health (Medical Education), a one year degree which around 12 students undertake per year (typically between years three and four of the medical course). Intercalating medical education students undertake modules in learning and teaching theory and practice, medical education research methods, assessment and appraisal, and curriculum development, carry out a medical education research project, and engage in medical education placement activities throughout their intercalated year. Placement activities include exposure to the work of the medical school admissions and partnerships and placement teams, a visit to another medical school with a different curriculum (problem-based), as well as teaching practice on campus and in our Clinical Practice Centre at St James’ Hospital. Most weeks, the intercalating medical education students contribute to the teaching of IDEALS on campus.

IDEALS (Innovation, Development, Enterprise, Leadership, and Safety) is the compulsory strand of the Leeds medical curriculum which was introduced to support medical students in their development to become professional doctors, in line with GMC expectations ( GMC, 2015, pp. 7-10). As the acronym suggests, IDEALS teaching addresses important themes such as patient safety, professionalism, leadership and teamwork, careers, learning and teaching skills, UK and international health care systems, and personal and professional development. Rather than producing homogenous doctors, IDEALS aims to encourage students to examine their own attitudes and beliefs and consider what type of doctor, teacher, team worker and leader they wish to be. Learning is primarily through group and individual reflection on the values, attitudes, and practices expected of a doctor.

IDEALS spans all years of the course and in year one, students are required to attend weekly small group sessions. Students remain in the same small group (n=12-13) and usually remain with the same tutor for the duration of years one and two. Tutors have varied experience, though most come from an educational or health professional background. In year one, learning is assessed through completion of reflective tasks for an e-portfolio, two in-course one-to-one meetings with the tutor to review progress, and questions in the integrated, end-of-year summative exam.

Intercalating medical education students teach first year IDEALS, working with the same group of year one students for the year alongside one of the university-employed IDEALS tutors. Medical education students typically start off observing the teaching, but become progressively involved in session design, delivery, and assessment at the discretion of the supervising tutor. IDEALS tutors provide ongoing feedback to the medical education students and assess their attendance, engagement, and contribution at two points in the year. This teaching therefore deviates from standard definitions of peer-assisted learning, which imply that the student teacher is entirely “non-expert” in teaching content and delivery ( Bennett et al., 2015, p. 607). Instead, our students are teaching content they are familiar with from their own IDEALS or equivalent study and have a growing understanding of educational theory and practice through their taught modules, supervision, assessment feedback, and ongoing written reflection on their practice. It is also important to note that medical education students are provided with IDEALS teaching materials and session plans, though they are encouraged to adapt and enhance these for the benefit of learners.

On reviewing relevant literature, we feel this example of near-peer teaching is distinctive in its longitudinal orientation and teaching topic (themes related to professionalism). A further distinguishing feature is that our near-peers are facilitating core sessions that are compulsory for first year students, rather than optional or additional sessions designed to reinforce learning. We therefore perceived it would be interesting and informative for others to read about our experiences and to gain new insights about the affordances of near-peer learning. This paper aims to bring together multiple perspectives on medical education student involvement in IDEALS teaching and to tease out aspects of our particular teaching context which support learning. The sections that follow contain reflections on the teaching from two medical education students (Lydia and Sonal, who was herself taught by a medical education student during her first year at medical school), a university-employed IDEALS tutor who supervises medical education students (Ken), a medical education graduate and now foundation year 2 doctor (Drew) and the medical education programme lead (Alison). First we reflect on the supportive features and challenges of our near-peer initiative, before discussing areas for development in near-peer teaching practice and scholarship.

## Supportive features of near-peer teaching in our context

In bringing our reflections together, we identified four key features of our particular near-peer teaching initiative: the valued perspective of more advanced students, regular ongoing near-peer contact, the ongoing support for near-peer tutors’ development, and the potential for near-peers to enhance the first year students’ learning experience. This section explains how these features benefit all parties involved, including year one medical students, intercalating medical education students, and IDEALS tutors who supervise medical education students.

### Valued perspective of more advanced students

Particularly in the early years of delivery, the course team were aware that there was a significant proportion of first year students who perceived IDEALS as less stimulating or challenging than other first year teaching. It was noted that some students struggled to engage, perhaps because they had too little clinical experience to understand the importance of topics such as professionalism, patient safety, and teamwork, or too little opportunity to build and reflect on their learning through practice. Drew was one of the first cohort to study IDEALS, before the intercalating medical education students became involved in near-peer teaching. Although he valued IDEALS’ emphases on communication and collaboration, there were times when he questioned its relevance:

..it did sometimes feel as though the sessions were straight out of a teamwork textbook or developed by course managers with little appreciation for the attitude of a first year student. With no clinical experience and therefore no obvious context, Friday morning sessions could turn into a very brutal ordeal for both students and tutor alike, especially if students perceived the session to be boring and of little relevance to them - despite the insistence of the tutor (Drew, medical education graduate).

One of the main contributions of near-peer teaching in our context is the medical education students’ support in emphasizing the clinical relevance of IDEALS. Year one students seem to value the perspective of their more experienced peers and medical education students seem to play a valuable role in recognising the needs of year one students and legitimising IDEALS study.

With an intercalated student co-facilitating the teaching, the learning and activities were specific, relatable and understandable enabling effective learning. Our medical education student often used her personal experiences of placements and clinical encounters to shape our learning, gave examples and provided insider knowledge of what was yet to come during our studies. As a near-peer, this was easier for us as students to relate to, a tutor far-removed from our studies may have found this more challenging (Sonal, previous first year student).

I was able to say how I remember a session being boring, and how at their stage I felt as if it was nonsense and a waste of everyone’s time. At the same time, by having completed a full year of placement in healthcare settings (and having completed three years of university), I was able to share real-life relatable experiences that emphasised the session theme: an example being getting shouted at by an intimidating consultant and thereafter discussing the barriers to speaking up when you feel that something is going wrong in an operation, and how to go about broaching it in a gentle way (Drew, medical education graduate).

These two reflections show how first year students value the perspective of medical education students, who are similar enough in age and experience to understand their needs and concerns, yet advanced enough to see the relevance of IDEALS for clinical practice. These reflections are reinforced by the feedback collected from first year students as part of standard university review processes. First year students’ comments about medical education students are consistently positive, with many pointing to the medical education students’ contribution in creating a collective learning culture, through which first year students are less afraid to share feelings and ideas and engage in discussion.

### Regular ongoing near-peer contact

Although medical education students tend to develop a rapport with their IDEALS group quite quickly, we have observed that near-peer relationships strengthen over time and that weekly contact fosters learning for first years and medical education students alike.

For the first years, a medical education student can seem more approachable than the university-employed IDEALS tutor and can become a trusted source of both academic and pastoral support. Previous first year students have reported that they appreciate the medical education students’ advice on adjusting to university life, preparing for exams, and medical careers, and value their encouragement and reassurance when things get tough. We have also noted the potential for medical education students to act as powerful role models and mentors, and for near-peer relationships to be sustained even after first year IDEALS has finished. This was particularly the case for Sonal, whose medical education tutor inspired her to intercalate in medical education herself:

Beyond IDEALS, we were able to discuss our academic work, career aspirations and receive tips and advice about medical school. Five years on, I am still in contact with the student who co-facilitated our sessions, she has graduated and now working as a foundation year 2 doctor. Being the first in my family to study medicine, this near-peer learning environment allowed me to develop connections with older students and therefore provided a platform for receiving careers guidance, academic support and someone to continually learn from. A further benefit I experienced from having a student tutor was the reassurance she could offer. Having experienced three years of medical school, she was reassuring and provided a realistic insight into medical school (Sonal, former first year student).

The university-employed IDEALS tutors have further observed the potential for near-peer relationships to develop over time, as evident in this reflection from Ken:

The value medical students place on co-facilitation and the presence of a near-peer is patently clear from the dynamics within the workshops week on week. Not only is there a tendency for higher levels of engagement and willingness to enter some very open and deepening discussions, but also, as the year progresses, to self-disclose more easily. By the end of the academic year, a strong collaborative culture is clearly palpable, with a firm, supportive social learning group, well prepared to progress to year 2. From an individual viewpoint, students transition from a relatively insecure position to one of a confident, well-placed individual. A principal reason for this is the presence of a BSc peer model who has travelled the road on which they have now embarked (Ken, IDEALS tutor and medical education placement supervisor).

Regular, ongoing contact also seems to be beneficial for medical education students’ development as educators. While first years are becoming more comfortable to disclose and seek support, the medical education students are becoming more comfortable in employing interactive teaching methods and asking for feedback on their teaching performance.

Working continually with the same group of students allowed us to build a good relationship and this meant that I was able to develop my teaching skills more so than working with different students. This was because I began to relax more throughout the year, students participated more during group work and students could give me feedback on how my teaching had improved (Lydia, medical education student).

The pedagogical style of IDEALS sessions was different to the teaching I had delivered before. It was a challenge to begin with to transition from a teacher to a facilitator - but the gradual improvement in my inquisitive questioning and gentle guiding of group discussion no doubt improved the learning experience of students. It also meant that my metacognitive skills developed, as I had to try and anticipate the answers that would be generated and the ensuing discussion. In practice since, I feel this has helped when exploring patients’ hidden agendas, and dealing with unexpected situations such as the sudden death of a patient and the discussion thereafter with distraught families (Drew, medical education graduate and foundation year two doctor).

As the lead for the intercalated medical education programme, Alison has observed how the requirement to teach the same group for a year is a strong motivating factor in encouraging medical education students to continue developing their practice:

By asking medical education students to teach the same IDEALS group for a year, they become more than an observer, visitor, or guest teacher. They are afforded *responsibility* for students’ learning and their teaching performance *matters.* As the medical education students will see the same group of students the following week, they are unable to simply forget about a previous teaching session and then move on uncritically. If a session doesn’t go well, the medical education students need to reflect on what could have been done better and identify an action plan for the following week. The medical students are supported in this process by the supervising IDEALS tutor, feedback on written reflections, as well as other parts of the medical education programme (Alison, intercalated medical education programme lead).

In our experience, medical education students experience a degree of pressure to perform when they are responsible for first years’ learning week on week and are teaching a compulsory part of the medical course.

### Ongoing support for near-peer tutors’ development

Unlike many of the peer-assisted learning initiatives reported in the literature ( Tai et al., 2016b; Yeung et al., 2017), our student-tutors are constantly developing their educational knowledge, skills, attitudes, and identities over a full academic year. Furthermore, they receive support for this development from their various medical education modules and their supervision from experienced IDEALS tutors. We see this as a strength of our near-peer initiative which is crucial to its success in supporting learning for all parties. Ken has described the valuable ways that he works with medical education supervisees:

Agreeing and planning the workshop sessions in advance, and encouraging pre-delivery briefings to ensure interactive social learning, is facilitated throughout and develops a progressive culture of enjoyable formative learning. Students are encouraged to participate in the in-course assessments and feedback, and attend the one-to-one meetings (with the consent of the Year 1 students) to gain an overview of student progress and an insight of verbal feedback on professionalism (Ken, IDEALS tutor and medical education placement supervisor).

In our context, the supervising IDEALS tutors play important roles in helping medical education students to prepare for sessions, assigning facilitation tasks as appropriate to the medical education student’s level and experience, ensuring that the intended learning outcomes of IDEALS are achieved, and providing feedback on medical education students’ teaching performance. Drew has highlighted the importance of the supervising tutors’ guidance for first years’ learning in IDEALS:

Near-peer tutors often have the best interests of their tutees at heart, yet it could be easy for them to underestimate the importance of a matter (as they too are still inexperienced) or to misinform tutees based on their assumptions. For this I feel it is important tutors are coached in emotional awareness and encouraged to reflect on their practice. The co-facilitation model of IDEALS means that there is a backstop in the form of a more experienced practitioner with whom you can seek clarity on uncertain issues. You could argue that removing this senior facilitator may allow the sessions to run more organically and to allow for greater openness between tutor and tutees - but we must remember there are objectives to be achieved and junior co-facilitators are still rather inexperienced in their trade - and there is a danger that sessions descend into a gossip session (Drew, medical education graduate and foundation year 2 doctor).

Though we are aware of contexts where it may be helpful for near-peers to teach more “organically” or independently ( Bennett et al., 2015; Stephens et al., 2016), we feel the guidance of an experienced tutor is essential when teaching important topics such as professionalism, patient safety, and teamwork; working with medical students in their first transitional year, and dealing with complex group dynamics. Although medical education students possess valuable insider experience of the medical course, they need help to ensure that first year students meet essential course requirements.

### Potential for near-peers to enhance the first year students’ learning experience

Although medical education students are presented with the IDEALS learning objectives and established teaching plans, they are increasingly encouraged to make the sessions their own by adapting content and methods and introducing new ideas. It is clear that the opportunity to contribute to the development of IDEALS is valued by medical education students, as well as the IDEALS tutors who volunteer to supervise the medical education placement:

We could adapt sessions and make them as interesting as possible for our students based on our own experiences. I think our input into making the sessions as interactive and enjoyable as possible was really appreciated by students. We could also amend teaching materials if we thought they were unclear, or related them more to a medical context. This was important as not all IDEALS tutors are from medical backgrounds (Lydia, medical education student).

Despite my many years teaching, I continue to learn much from intercalating MedEd students. There are opportunities for them to be innovative and create pedagogic products at the end of the academic year which can be a legacy for future Year 1 students. A recent example was a medical education student’s co-creation of a Mindfulness Video to facilitate student learning around ways of managing stress levels during intensive periods of study (Ken, IDEALS tutor and medical education placement supervisor).

This is just one of several examples of ways in which medical education students have contributed to IDEALS review processes. These contributions lead us to believe that there is added value in partnering employed tutors with medical education students and that medical education student involvement in IDEALS should be sustained.

## Challenges of near-peer teaching in our context

As with any teaching intervention, our near-peer initiative is not without its challenges. Two particular challenges for medical education students will be described here: establishing the role of the near-peer and taking on the role of co-tutor.

### Establishing the role of the near-peer

As part of their education placement module, medical education students are required to keep a reflective journal recording their learning and progress throughout the year. Within this journal, students commonly reflect on the challenges of adopting a tutor role, such as taking the lead when required, facilitating discussion rather than “talking at” the first years, maintaining boundaries, and feeding back on poor performance. It takes medical education students time to understand their near-peer role and to convey appropriate levels of authority and professionalism.

The main challenge for me was learning where to position myself on the scale from peer to teacher. Teaching IDEALS as a medical education student is a unique situation because we are teaching as part of our placement module and are assessed for our own degree, yet we are also peers completing the same course as the students, without any official title. Therefore knowing how formal to be was challenging. An example of this was when students asked for exam advice - as a peer I wanted to give very pragmatic advice, yet some of this advice wouldn’t be what a teacher would say and could be deemed unprofessional. In addition, when students were talking amongst themselves when they should have been listening to me, my co-facilitator or another student, it was difficult to “discipline” them. I personally just asked them to be quiet and was confident doing so, but I am aware that other medical education students found this more challenging. Interestingly, on one occasion, my co-facilitator did ask two students to move apart, something that I would not have felt able to do (Lydia, medical education student).

As reported in the peer-assisted learning literature ( Tai et al., 2016a), a particular challenge for the medical education students is giving feedback to their students. This seems to be a critical tipping point, where the balance can shift from peer towards teacher.

It became challenging during assessments where I was asked to feedback to the students on their performance and professionalism. I felt uneasy about being honest with my opinion as I was worried it could ruin the rapport build. It was here where I realised the importance of maintaining my professional boundaries. It took time and experience for me to develop the confidence to give students constructive feedback from which they could develop and learn from in the future (Sonal, medical education student).

Supervising tutors can be helpful to medical education students in discussing the challenges of assessment, but ultimately the task of establishing a near-peer role is not clear cut, nor easily resolved by the end of the year-long placement. It is hoped that this experience of role negotiation is beneficial to the medical education students in giving them an insight into the challenges of developing an educator identity and the life-long endeavour of developing one’s educational practice.

### Taking on the role of co-tutor

Most of the time, medical education students thrive on this educational opportunity and develop supportive relationships with their IDEALS supervisor and year one students. However, there are rare occasions when a medical education student struggles to develop an effective working relationship with the staff tutor and student group. This may be because of personality differences, different teaching perspectives or expectations, or a lack of confidence or initiative in taking on the tutor role.

A memorable example was when I received complaints about a medical education student from an IDEALS tutor, who was concerned that the student was not responding to feedback on her performance, nor submitting teaching plans as requested. When I met with the student concerned, it transpired that the student was questioning her entitlement to teach the first year students and lacked the confidence to suggest changes to the teaching material. I then reminded the student that she was not expected to “know” everything and that her greatest contribution to IDEALS was her experience as a student to date. I emphasized the importance of asking for help and meeting agreed deadlines for session plans, so that she and her co-tutor could support each other in designing and delivering a valuable learning experience for first years. I also spoke to the tutor about reinforcing these messages and recognising any contributions the student made. We are also now looking into ways that medical education students could observe a range of IDEALS tutors, to help them in identifying and reflecting on different teaching perspectives (Alison, medical education programme lead).

This last reflection shows how traditional understandings of knowledge and expertise can be persistent in medicine, and can prevent medical education students from reaching their potential as effective facilitators of learning. As the years have passed, we have introduced additional orientation sessions for the medical education students to further promote collaborative understandings of teaching and learning in IDEALS. It is possible that one of the main benefits of IDEALS for medical education students is the challenge it poses to previous understandings of learning, as evident in Drew’s reflection on the initiative:

As near-peer tutors, we do not know all of the answers all of the time: this does not prohibit us from doing our job but merely emphasises the importance of being honest, asking for help, acknowledging our limits and the need for ongoing learning. These behaviours are crucial for a successful career in medicine (Drew, medical education graduate and foundation year 2 doctor).

Reflections such as these have prompted us to explore the later teaching and learning experiences of medical education graduates and we are starting to develop research in this area.

## Discussion

In contrast to other near-peer teaching described in the literature, our near-peers are teaching themes around professionalism, teaching essential rather than optional or additional components of the medical course, teaching a consistent group of students, and developing as educators over a full academic year. This appears to influence the near-peer relationship in complex and interesting ways, as we hope we have alluded to in this paper.

Previous definitions of peer-assisted learning have emphasized the non-expert status of students as teachers, and peer teachers are understood to be neither content nor teaching experts ( Bennett et al., 2015; Ross & Cameron, 2007). However, in our context, near-peer teachers are much valued for their expertise. Although the employed IDEALS tutors contribute more initially in terms of teaching expertise, medical education students are considered experts of the Leeds medical course, undergraduate medical study, and early clinical experience, and are appreciated by first years for the tips and experiences they share. Furthermore, as medical education students, our near-peers are continually developing expertise in educational theory and practice. The expertise of both tutors (staff and student) intersect to provide rich interactive learning experiences for all concerned. It is therefore misleading to think of our near-peers as entirely non-expert, particularly when they have devoted a full year to developing their teaching.

Likewise, previous authors have explained benefits of peer-assisted learning in terms of the high level of congruence between teachers and learners ( Ross & Cameron, 2007; Stephens et al., 2016). Indeed, there is some evidence in our setting to suggest that first year medical students see medical education students as “one of us”. However, recent papers have started to challenge ideas around congruence and have drawn attention to the ways that understandings of the role of teachers as experts persist. In Bennett et al.’s (2015) activity systems analysis of student-led case-based discussions in clinical practice, students rejected the roles of peer teacher, student, and evaluator, as they saw the teacher role as a “claim to expertise” (p. 605). In contrast, our medical education students tend to aspire to take on a more expert role. Though this is clearly challenging for them, medical education students work hard to establish authority through “taking the lead”, “maintaining boundaries”, and giving honest and “constructive feedback”. These behaviours are reinforced by the tutors supervising them and we have noted that medical education students struggle when they take on a more passive role within sessions. Despite the emphasis on collaborative learning within IDEALS, it seems that *all* parties still expect the teacher to be authoritative to an extent and that there may be less difference between near-peer and university-employed tutors than one would expect. This observation is similar to Cianciolo et al.’s (2016) finding that variation was greater within than across tutor types (near-peer vs university staff), and indicates that there may be some equally fascinating contradictions in our context.

It is interesting to note that in IDEALS, medical and clinical experience seems to be valued by first year students most. Furthermore, the perspective of students who are slightly more advanced in their training can sometimes be privileged over the perspective of a university-employed IDEALS tutor who is much more experienced at medical teaching. Perhaps this is because medical education students are seen to belong to the community that first year students are working to become part of, albeit only slightly closer to the centre of medical practice than themselves. Though university-employed IDEALS tutors are respected for their expertise in teaching, they are not necessarily part of the community the first years aspire to join and for this reason, may not be valued in the same way. This leads us to consider whether Communities of Practice theory ( Lave and Wenger, 1991) may be more helpful than notions of social and cognitive congruence in understanding near-peer teaching in our context. Communities of Practice theory may further be useful in understanding the medical education students’ development as educators through co-facilitation with a more experienced tutor, and the potential for both medical education students and university-employed IDEALS tutors to act as role models for less experienced students.

IDEALS, with its themes around professionalism, leadership, communication, and teamwork, in many ways is the ideal arena for medical education students to be developing their practice and identities as educators. Medical education students are able to contribute to IDEALS teaching relatively quickly, as they can begin by sharing examples and advice based on their own experience. The interactive, small-group nature of IDEALS teaching allows the medical education students to develop their facilitation skills with support, guidance, and feedback from an experienced IDEALS tutor. The regular weekly format of IDEALS means that medical education students can develop their near-peer role over time, develop strong and lasting relationships with first year students, and are highly motivated to improve their teaching practice. As IDEALS arguably relies less on text-book knowledge than other first year modules, there is also plenty of scope for medical education students to make meaningful changes and additions to the teaching. These experiences are no doubt helpful for medical education students’ developing educator identities.

In preparing this paper, it has been enlightening to bring together the reflections of previous first year and medical education students, including one graduate who is now a foundation year two doctor. It has been rewarding to learn how medical education students have inspired first year students and to consider the ways that IDEALS teaching has informed medical graduates’ later teaching in clinical practice. It was beyond the scope of this paper to explore long-term outcomes in any depth, however this is something we would like to research in the future. Based on work undertaken for this paper, we predict observational methods and socio-cultural theoretical perspectives will be key to understanding medical education students’ ongoing learning in our context.

## Conclusions

Peer-assisted learning initiatives vary greatly in terms of teaching topics, the teachers and learners involved, the location of teaching, and the amount of training student-tutors receive. In this paper we have reflected on a near-peer initiative, which involves medical education students in the teaching of fundamental aspects of professionalism to first year students on campus. Features which appear to support learning in this context include the valued perspective of more advanced students, regular ongoing near-peer contact, the ongoing support for near-peer tutors’ development, and the potential for near-peers to enhance the first year students’ learning experience. A range of stakeholders’ reflections have revealed particular challenges faced in this context, and have highlighted contradictions in the ways that the near-peer’s role is understood. Further research is needed to understand these contradictions and the ways that students and tutors negotiate different understandings of teaching and learning. Observational methods and socio-cultural theoretical perspectives should be helpful in this regard.

## Notes On Contributors

Dr Alison Ledger leads the BSc in Applied Health (Medical Education) programme, the intercalated medical education degree at the University of Leeds. She is an active researcher within the Leeds Institute of Medical Education, with a particular interest in how individuals and teams learn to work in changing contexts.

Lydia Edwards and Sonal Mistry are graduates of the BSc in Applied Health (Medical Education) programme, who have now returned to the 4th year of the medical course at Leeds.

Dr Drew Harding is a graduate of the BSc in Applied Health (Medical Education) and MBChB programmes at Leeds. He is now in his second foundation year as a junior doctor.

Ken Hargreaves is a tutor at the University of Leeds, responsible for facilitating IDEALS teaching in the MBChB and PG Physician Associate programmes and supervising intercalating medical education students on placement.

## References

[ref1] Bennett D. O’Flynn S. & Kelly M. (2015). Peer assisted learning in the clinical setting: an activity systems analysis. Advances in Health Sciences Education. 20,595–610. 10.1007/s10459-014-9557-x 25269766 PMC4495258

[ref2] Cianciolo A. T. Kidd B. & Murray S. (2016). Observational analysis of near-peer and faculty tutoring in problem-based learning groups. Medical Education. 50(7),757–767. 10.1111/medu.12969 27295480

[ref3] General Medical Council.(2014). Good medical practice. Manchester, UK: GMC.

[ref4] General Medical Council.(2015). Outcomes for graduates (Tomorrow’s Doctors). Manchester, UK: GMC.

[ref5] Irvine S. Williams B. & McKenna L. (2017). How are we assessing near-peer teaching in undergraduate health professional education? A systematic review. Nurse Education Today. 50,42–50. 10.1016/j.nedt.2016.12.004 28012361

[ref6] Lave J. & Wenger E. (1991). Situated learning: Legitimate peripheral participation. Cambridge: Cambridge University Press. 10.1017/CBO9780511815355

[ref7] Ross M. T. & Cameron H. S. (2007). Peer assisted learning: a planning and implementation framework: AMEE Guide no. 30. Medical Teacher. 29(6),527–545. 10.1080/01421590701665886 17978966

[ref8] Stephens J. R. Hall S. Andrade M. G. & Border S. (2016). Investigating the effect of distance between the teacher and learner on the student perception of a neuroanatomical near-peer teaching program. Surgical & Radiologic Anatomy. 38(10),1217–1223. 10.1007/s00276-016-1700-3 27225186 PMC5104784

[ref9] Tai J. H. Canny B. J. Haines T. P. & Molloy E. K. (2016a). The role of peer-assisted learning in building evaluative judgement: Opportunities in clinical medical education. Advances in Health Sciences Education. 21(3),659–676. 10.1007/s10459-015-9659-0 26662035

[ref10] Tai J. Molloy E. Haines T. & Canny B. (2016b). Same-level peer-assisted learning in medical clinical placements: a narrative systematic review. Medical Education. 50(4),469–484. 10.1111/medu.12898 26995485

[ref11] Yeung C. Friesen F. Farr S. Law M. & Albert L. (2017). Development and implementation of a longitudinal students as teachers program: participant satisfaction and implications for medical student teaching and learning. BMC Medical Education. 17:28. 10.1186/s12909-017-0857-8 28143483 PMC5282841

