# Supplementary material for: Learning ideals: Critical reflections on a near-peer initiative at Leeds
Source: MedEdPublish (2016). 2017 Sep 13;6:157. [Version 1] doi: 10.15694/mep.2017.000157 (PMC10885291; doi:10.15694/mep.2017.000157)
Supplement: Peer review history

# Reviewer report: Reviewer response for version 1

**DOI:** 10.21956/mep.19346.r28275

**Published:** 2017-09-14

**Authors:** Julie Browne

**Affiliations:** 1 Cardiff University School of Medicine

This review has been migrated. The reviewer awarded 4 stars out of 5 Thank you for a very interesting and stimulating article. I enjoyed reading it and, as programme lead for the intercalated degree in medical education at my own institution where we also involve students in PAL, I recognised a great number of the joys and challenges of organising and engaging in a programme of peer to peer learning.There are two aspects of this paper that particularly impressed me. First, I was impressed by the diverse team of co-authors, all of whom clearly worked well together as a group and had something constructive and unique to add to the discussion as individuals. This, of course, is a model of how good peer learning works in action.Secondly, I was struck by the ambition of the enterprise and the fact that the students appear to have undertaken it with enjoyment and success. Medical students have always shared learning about clinical skills and factual material with each other, both formally and informally - probably since the days of Hippocrates! But you have chosen to take on the far more difficult task of involving students in the teaching of skills related to personal and professional development over a whole year; as you suggest, this presents an altogether more challenging context. Your collective reflections on this are therefore particularly interesting as they indicate that medical students probably are under-utilised as teaching faculty. Your conclusions suggest that we are currently only scratching the surface of the potential that peer to peer teaching and learning can offer and, as yet, we have insufficiently robust analyses of whether (and if so, how far, by what means, and why) it is truly successful in achieving its intended outcomes. I heartily agree and therefore hope you will continue this very interesting line of work.

Reviewer Expertise:

NA

No decision status is available

# Reviewer report: Reviewer response for version 1

**DOI:** 10.21956/mep.19346.r28274

**Published:** 2017-09-13

**Authors:** Trevor Gibbs

**Affiliations:** 1 AMEE

This review has been migrated. The reviewer awarded 5 stars out of 5 I very much enjoyed reading this paper and wish to recommend it whole-heartedly to all those involved in curricula planning. I learned much from this paper and how it took an underlying philosophy ( IDEAL) and interpreted it and its principles into an approach ( peer-peer and near-peer learning) , which as the authors suggest still needs to be exposed to more research to prove its value beyond that of just improving knowledge transfer.An issue that we all have to face is the competition between service, research and teaching activities and we need to find effective ways of facilitating student learning within this competitive environment. The final element of this research I think is its long term effects upon our future educationalists and our practitioners of the future.

Reviewer Expertise:

NA

No decision status is available
